# Neuroactive Steroids in First-Episode Psychosis: A Role for Progesterone?

**DOI:** 10.1155/2016/1942828

**Published:** 2016-09-22

**Authors:** Martino Belvederi Murri, Flaminia Fanelli, Uberto Pagotto, Elena Bonora, Federico Triolo, Luigi Chiri, Fabio Allegri, Marco Mezzullo, Marco Menchetti, Valeria Mondelli, Carmine Pariante, Domenico Berardi, Ilaria Tarricone

**Affiliations:** ^1^Department of Neurosciences, Rehabilitation, Ophthalmology and Genetics, Section of Psychiatry, University of Genova, Genova, Italy; ^2^Department of Psychological Medicine, King's College London, London, UK; ^3^Section of Endocrinology, Department of Medical and Surgical Sciences and Center for Applied Biomedical Research (CRBA), St. Orsola-Malpighi Hospital, University of Bologna, Bologna, Italy; ^4^Bologna Transcultural Psychosomatic Team (BoTPT), Department of Medical and Surgical Sciences, University of Bologna, Bologna, Italy; ^5^Section of Medical Genetics, Department of Medical and Surgical Sciences, University of Bologna, Bologna, Italy; ^6^Department of Medical and Surgical Sciences, University of Bologna, Bologna, Italy

## Abstract

Neuroactive steroids may play a role in the pathophysiology of psychotic disorders, but few studies examined this issue. We compared serum levels of cortisol, testosterone, dehydroepiandrosterone, and progesterone between a representative sample of first-episode psychosis (FEP) patients and age- and gender-matched healthy subjects. Furthermore, we analyzed the associations between neuroactive steroids levels and the severity of psychotic symptom dimensions. Male patients had lower levels of progesterone than controls (*p* = 0.03). Progesterone levels were inversely associated with the severity of positive symptoms (*p* = 0.007). Consistent with preclinical findings, results suggest that progesterone might have a role in the pathophysiology of psychotic disorders.

## 1. Introduction

Preliminary evidence points to a possible role of several neuroactive steroids in the pathophysiology of psychotic disorders, but clinical evidence is still scarce. Neuroactive steroids such as cortisol (CORT), progesterone (PROG), testosterone (T), dehydroepiandrosterone (DHEA), and estrogens play an important role in shaping the* central nervous system (CNS)* structure and function throughout the lifespan [1]. Neuroactive steroids modulate a wide array of functions in the brain, spanning from the neurodevelopment to adult neurogenesis [2] and cognition [3]. In adult life, they exert a significant influence on the activity of several neurotransmitter systems that are relevant to the pathophysiology of psychosis, such as the dopaminergic [4], glutamatergic, and GABAergic systems [5].

In the past years, most studies on the role of neuroactive steroids in psychosis have focused on cortisol, which is the main end product of the Hypothalamic-Pituitary-Adrenal (HPA) axis. A large body of evidence is now available indicating that the dysregulation of the HPA axis activity plays a detrimental role in the vulnerability to and in the course of psychotic disorders [6–8]. In addition, epidemiological and neurobiological data suggest that the administration of exogenous estrogens might alleviate psychotic symptoms in patients with established schizophrenia of both genders [9, 10].

More recently, preclinical studies have provided hints that other neuroactive steroids might be relevant to the pathophysiology of psychosis and lead to possible therapeutical applications. These include DHEA, T, and PROG. Of note, the administration of DHEA [11], the modulation of androgen-related pathways [12], and the administration of PROG [13–15] exerted antipsychotic-like effects in animal models of schizophrenia. Despite this promising preliminary evidence, we still lack evidence on the status of neuroactive steroids in patients with psychotic disorders. Few studies investigated the circulating levels of PROG in patients with psychosis and yielded conflicting results. Levels of PROG were higher than [16], similar to [17–19], or lower than [20] those of healthy controls. Similarly DHEA levels were found to be lower than, higher than, or similar to controls [11], whereas levels of T were lower [18, 21] or normal [16, 22]. Moreover, only few studies examined if there were associations between these hormone levels and the severity of psychopathology [23]. Notably, the interpretation of these findings needs to take into account the confounding effects of pharmacological treatments, of the illness progression and endocrine-metabolic changes that occur in patients along the different stages of the illness. This study aims to assess the circulating levels of various neuroactive steroids, namely, CORT, DHEA, T, and PROG, in a group of* males and females* at their first episode of a psychosis and age- and gender-matched healthy controls, while testing the presence of associations between hormone levels and severity of psychotic symptoms.

## 2. Methods

### 2.1. Subjects

The study was conceived as an add-on to the European Network of National Schizophrenia Networks Studying Gene-Environment Interactions (EU-GEI) study for the Bologna Centre, Italy [24]. We approached all subjects with FEP who were consecutively treated in the mental health services of the catchment area of the Bologna EU-GEI centre between January 2011 and November 2013. Inclusion criteria were age 18–64 and first episode of functional psychotic illness. Once patient's clinical conditions were stable to provide informed consent, he/she was asked to participate in our study and donate blood for additional hormonal level analyses. Patients underwent clinical and physical examinations including ECG and routine laboratory tests to exclude physical illness.

Healthy controls were selected from a population that was examined in a recent study, aimed at establishing normative levels of circulating steroidal hormones [25]. Data regarding sociodemographic, clinical characteristics and psychiatric comorbidities were available from 416 healthy subjects aged 18–64 years, living in a nearby catchment area of the cases. From this sample, we further excluded subjects who were suffering from relevant physical disorders, including endocrinological disorders, obesity (BMI > 30), psychiatric illnesses, and substance abuse, and those who were taking any medications. Those with clinically relevant depressive symptoms (a score of 20 or higher at the Beck Depression Inventory) were also excluded to avoid confounding effects on HPA axis activity. This led to a subsample of 153 participants, from which we randomly selected age-, gender-, and BMI-matched controls. The protocol was approved by the local Ethics Committee, in accordance with the Declaration of Helsinki.

### 2.2. Assessment

Patients with FEP were assessed by experienced psychiatrists. Validation of the clinical diagnosis on DSM-IV criteria was obtained by researchers trained in the use of Operational Criteria (OPCRIT) [26]. During the same week of the blood drawing, patients were evaluated for the severity of their clinical symptoms using the positive and negative syndrome scale (PANSS). From PANSS scores, the values of five symptom dimensions (negative symptoms, disorganization, positive symptoms, excitement, and anxiety/depression) were calculated [27]. The DUP was assessed using the Nottingham Onset Schedule [28]. Exposure to antipsychotics (AP) was converted in chlorpromazine equivalents, according to recent indications [29].

### 2.3. Hormone Assays

Five millilitres of blood was collected between 0800 and 1000 from fasting subjects using Vacuette Z serum beads clot activator tubes (Greiner Bio-One, Kremsmünster, Austria). Subjects were asked to abstain from physical activity, caffeine consumption, and smoking on the morning of the assessment. Among females, the hormonal sampling was carried out in the follicular phase of the menstrual cycle for all subjects (range 5–12 days). Samples were centrifugated at 2000 ×g for 10 min at room temperature and sera stored in 1.5 mL polypropylene tubes at −20°C until analysis. The circulating levels of CORT, T, DHEA, and PROG were assessed from serum by isotopic dilution-liquid chromatography tandem mass spectrometry (ID-LC-MS/MS), following the protocol described in a recent study [25].

### 2.4. Data Analyses

First, the normality of data was assessed using a Shapiro-Wilk test. Then, levels of hormones were compared between cases and controls using a matched paired *t*-test or Wilcoxon test as appropriate. We also ran exploratory correlation analyses (Pearson's *r* or Spearman's rho, as appropriate) to examine the associations between hormonal levels and other factors, including the severity of symptoms. If significant associations were detected, we subsequently adjusted for the severity of other symptom dimensions in multiple linear regression models to account for their potential confounding effect [7]. SPSS 15.0 was used for all analyses.

## 3. Results

Among 47 patients who were approached, 32 subjects (17 males, 15 females) met inclusion criteria and accepted to take part in the study.  Table 1 reports data on participants' characteristics. Nine patients were hospitalized at the moment of the hormonal evaluation; one was drug-naive, 6 were receiving quetiapine, 9 were receiving risperidone, 8 were receiving olanzapine, 2 were receiving aripiprazole, 5 were receiving oral haloperidol, and 1 was receiving clozapine.

Males with FEP had lower levels of CORT (*p* = 0.03) and PROG (*p* = 0.03) than healthy male controls, while differences were not significant for levels of T (*p* = 0.74) and for DHEA (*p* = 0.09). In contrast, we did not find significant differences in hormonal levels between female patients and female controls.

In exploratory analyses, we did not find significant correlations between the hormonal levels and the cumulative dose of AP treatment, the duration of AP treatment, and its mean daily dose, except for T (daily dose: *r* = 0.40, *p* = 0.02). Also, recent use of cannabis was not associated with the levels of any neuroactive steroids in either gender or in the whole sample.

Regarding the association with clinical symptoms, among males, the levels of PROG were inversely associated with the severity of positive symptoms (Spearman's rho = −0.67, *p* = 0.007) but not with other symptom dimensions (all *p* > 0.18). This association held after adjusting for the other symptom dimensions in linear regression (*p* = 0.02). Instead, levels of CORT, DHEA, and T were not associated with the severity of any symptom dimension. Among females, levels of DHEA correlated positively with negative symptom dimension (*r* = 0.51, *p* = 0.049) and disorganization (*r* = 0.56, *p* = 0.03). Also, progesterone levels correlated with disorganization (*r* = 0.59, *p* = 0.02). However, none of these associations remained statistically significant after adjusting for other symptom dimensions in regression models.

## 4. Discussion

We found that male patients with first-episode psychosis had lower levels of progesterone than those of age- and gender-matched healthy controls. Furthermore, the levels of progesterone correlated negatively with the severity of positive symptoms, lending support to a possible role of this hormone in the pathophysiology of psychotic disorders. To our knowledge, this study was the first to investigate the levels of various neuroactive steroids among patients with a first episode of psychotic illness, not restricted to schizophrenia, and with a relatively low exposure to antipsychotics.

The evidence on progesterone levels in psychotic disorders is still scarce; however our results are consistent with those obtained in another study on male, antipsychotic-naive patients with schizophrenia, during later episodes of their illness [20]. Again, in another recent study on bipolar disorder, males (but not females) who displayed positive psychotic symptoms had lower levels of PROG compared with those who did not [30]. Furthermore, data from the former study showed that the levels of PROG tended to increase following treatment with AP, but they were still lower than those of healthy controls at the end of follow-up [20]. Nevertheless, other studies conducted on males with schizophrenia, receiving antipsychotic treatment of longer duration, did not find significant differences in PROG levels with controls [17, 19, 31]. Consistently, the increase of the adrenal production of progesterone induced by some AP drugs (olanzapine and clozapine) was found to mediate part of their pharmacological efficacy in animal models [32]. Taken together, these evidences support the view that lower levels of PROG in males with psychotic disorders could persist after initial antipsychotic drug therapy but normalize in later stages of the illness.

Our results, instead, are in apparent contrast with those of other studies finding elevated levels of both PROG and CORT among antipsychotic-naive, acute-phase patients with schizophrenia [16, 33]. However, Guest and colleagues did not differentiate between genders; thus results cannot be directly compared with ours. Also, PROG, similarly to CORT, can display stress-related increases [34]; thus increases of its levels might reflect state- rather than trait-related fluctuations. In our study, in fact, CORT was also reduced in male patients compared to controls, which could be due to the measurement of the hormone during the stable phase of the illness [7], especially after treatment with predominantly second-generation agents [35]. Lastly, it must be acknowledged that differences in the diagnostic composition of the sample* or other characteristics* may also have played a role.

Clearly, further studies are needed to corroborate and extend these findings; however, they suggest that PROG levels could be altered in male patients with first-episode psychosis, and this might contribute to a vulnerability towards positive symptoms. Biological plausibility is based on several lines of evidence: PROG can easily cross the blood-brain barrier and exert anxiolytic and neuroprotective actions, mainly via its conversion to the neurosteroid allopregnanolone and subsequent interactions with several neurotransmitters systems, including GABA and dopamine [34, 36]. Hence, a relative deficiency of this hormone might increase the likelihood of dysregulation in specific neurotransmitter systems that are involved in the pathogenesis of psychotic symptoms [37, 38]. Conversely, preclinical evidence showed that the administration of exogenous PROG could exert antipsychotic-like effects [14, 15].* Lastly, recent evidence suggests that antipsychotics benefits might depend on their anti-inflammatory role [39]: interestingly, progesterone may share similar properties [40].* Further research should clarify the status of PROG in psychotic disorders, with the ultimate aim of possible therapeutical applications [13].

To our knowledge, this was the first study investigating the levels of PROG among patients with FEP, not limited to the diagnosis of schizophrenia. We carefully matched a representative sample of patients with FEP with healthy controls and measured the hormone levels with a reliable method [25]. However, these findings should be considered as preliminary and need further confirmation in light of some limitations. First of all, the sample size was relatively small* and clinically heterogeneous* and might have been underpowered to detect meaningful associations.* Also, the use of different antipsychotic drugs or other subject characteristics may have played a confounding role that at present we cannot rule out.* Nonetheless, the association between PROG levels and positive symptoms held after adjusting for confounding effects for other symptom dimensions. Second, we did not measure prolactin levels. However, no patient showed signs or symptoms of hyperprolactinemia at the time of the exam, and previous studies showed no association between progesterone and prolactin in males [19]. Third, we did not control for smoking status, although cigarette smoking does not seem to affect most of the hormones we analyzed [41]. We acknowledge that future studies should include this information.* Lastly, we have not corrected analyses for multiple comparisons, given the reduced sample size and pilot/exploratory nature of this study.*

## 5. Conclusions

In conclusion, we found that progesterone was reduced in male but not female patients with a first episode of psychotic disorders, and it was negatively correlated with the severity of positive symptoms of psychosis. Whether it has a role in the vulnerability to psychotic disorders or it is merely an epiphenomenon of an altered stress response, it remains to be settled; nonetheless extending the knowledge on the status and role of PROG in psychosis might contribute to the study of novel therapeutical applications.

## Figures and Tables

**Table 1:** Comparison between cases and controls.

	Males			Females		
	Cases (*n* = 17)	Controls (*n* = 17)	Statistics^a^	Cases (*n* = 15)	Controls (*n* = 15)	Statistics
Age	29.5 ± 10.5	30.3 ± 10.5	*F* = 0.19, *p* = 0.85	31.2 ± 7.1	32.2 ± 6.0	*F* = 0.42, *p* = 0.68
BMI	23.7 ± 4.0	24.4 ± 3.3	*F* = 0.53, *p* = 0.60	22.5 ± 3.6	23.1 ± 3.3	*F* = 0.44, *p* = 0.66
Recent cannabis use, %	41.2	0	*χ* ^2^ = 8.82, *p* = 0.003	26.7	0	*χ* ^2^ = 4.62, *p* = 0.03^*∗*^
DUP, weeks	119 (42–424)	—		42 (14–90)		
Days of AP treatment	62 (17–149)	—		146 (27–179)		
Mean daily dose of AP^b^	376 (211–571)	—		274 (83–323)		
Diagnosis, %						
*Schizophrenia*	35.3	—		33.3	—	
*Bipolar disorder *	5.9	—		6.7	—	
*Major depression*	11.8	—		6.7	—	
*Other* ^c^	47.1			53.3	—	
PANSS, total score	64.6 ± 17.2	—		64.1 ± 18.8		

Hormonal levels^d^						
CORT	120.6 (40.7)	143.1 (38.2)	*F* = 2.36, *p* = 0.03^*∗*^	120 (100–158)	130 (77–138)	*z* = 0.34, *p* = 0.73
T	5.30 (1.48)	5.12 (1.79)	*F* = 0.34, *p* = 0.74	0.22 (0.16–0.28)	0.26 (0.18–0.32)	*z* = 0.68, *p* = 0.50
DHEA	6.67 (3.73)	8.67 (5.82)	*F* = 1.83, *p* = 0.09	3.93 (3.03–6.94)	5.68 (4.78–7.73)	*z* = 1.14, *p* = 0.26
PROG	0.05 (0.03–0.10)	0.10 (0.08–0.12)	*z* = 2.15, *p* = 0.03^*∗*^	0.06 (0.03–0.42)	0.18 (0.09–0.43)	*z* = 1.19, *p* = 0.23

^a^Data are compared using the paired-samples *t*-test if normally distributed (mean ± standard deviation reported) or with the paired-samples Wilcoxon test if skewed median (interquartile range reported); ^b^chlorpromazine equivalents; c includes brief psychotic disorder, psychotic disorder not otherwise specified, and delusional disorder; ^d^unit of measure: nmol/L.

BMI: body mass index; DUP: duration of untreated psychosis; AP: antipsychotics; PANSS: positive and negative syndrome scale; CORT: cortisol; T: testosterone; DHEA: dehydroepiandrosterone; PROG: progesterone.

^*∗*^ *p* < 0.05.
